# Effect of Pressure, Reconstituted RTE Meat Microbiota, and Antimicrobials on Survival and Post-pressure Growth of *Listeria monocytogenes* on Ham

**DOI:** 10.3389/fmicb.2018.01979

**Published:** 2018-08-27

**Authors:** Januana S. Teixeira, Lenka Repková, Michael G. Gänzle, Lynn M. McMullen

**Affiliations:** Department of Agricultural, Food and Nutritional Science, University of Alberta, Edmonton, AB, Canada

**Keywords:** high pressure processing, nisin, *Listeria monocytogenes*, *Leuconostoc gelidum*, Lactobacillus sakei, meat microbiota, antimicrobials, ready-to-eat meat

## Abstract

Pressure treatment of ready-to-eat (RTE) meats extends the shelf life and reduces risks associated with *Listeria monocytogenes*. However, pressure reduces numbers of *Listeria* on ham by less than 5 log (CFU/g) and pressure effects on other meat microbiota are poorly documented. This study investigated the impact of pressure and RTE meat microbiota, with or without nisin and rosemary oil, on survival of *Listeria* after refrigerated storage. Ham was inoculated with a 5-strain cocktail of *L. monocytogenes* alone or with a cocktail of RTE meat microbiota consisting of *Brochothrix thermosphacta*, *Carnobacterium maltaromaticum*, *Leuconostoc gelidum*, and *Lactobacillus* *sakei*. Products were treated at 500 MPa at 5°C for 1 or 3 min, with or without rosemary extract or nisin. Surviving cells were differentially enumerated after pressure treatment and after 4 weeks of refrigerated storage. After 4 weeks of storage, products were also analyzed by high throughput sequencing of 16S rRNA amplicons. Pressure treatment reduced counts of *Listeria* by 1 to 2 log (CFU/g); inactivation of RTE meat microbiota was comparable. Counts of *Listeria* increased by 1–3 log (CFU/g) during refrigerated storage. RTE meat microbiota did not influence pressure inactivation of *Listeria* but prevented growth of *Listeria* during refrigerated storage. Rosemary extract did not influence bacterial inactivation or growth. The combination of nisin with pressure treatment for 3 min reduced counts of *Listeria* and meat microbiota by >5 log (CFU/g); after 4 weeks of storage, counts were below the detection limit. In conclusion, pressure alone does not eliminate *Listeria* or other microbiota on RTE ham; however, the presence of non-pathogenic microbiota prevents growth of *Listeria* on pressure treated ham and has a decisive influence on post-pressure survival and growth.

## Introduction

Processing of packaged RTE meats products with high pressure is used by the meat industry to eliminate *Listeria monocytogenes*. Pressure processing does not alter the quality of RTE meats quality and is thus considered an attractive alternative to chemical preservatives. High pressure treatment disrupts bacterial membranes, and when used after packaging can be an effective method to reduce the overall microbial load and to extend the storage life of RTE products (Chen et al., 2012). Pressure treatment of RTE meat does generally not achieve a 5 log reduction of cell counts of *L. monocytogenes*, the most significant food safety concern associated with RTE meats. Treatment at 450 MPa reduced cell counts of *L. monocytogenes* by 1–3 log CFU/g (Chung et al., 2005; Morales et al., 2006). Treatments on ham at 500 or 600 MPa reduced cell counts by 3–4 log (CFU/g) when the product was tempered to refrigeration temperature prior to pressure treatment (Teixeira et al., 2016). Moreover, the decrease in microbial viability after pressure treatments is partially compensated by recovery of sublethally injured *L. monocytogenes* during post-pressure refrigerated storage (Bull et al., 2005; Marcos et al., 2008; Juck et al., 2012; Muñoz-Cuevas et al., 2013; Teixeira et al., 2016).

*Listeria monocytogenes* can recover even after the application of more than 600 MPa, the upper pressure limit of current commercial equipment for pressure treatment (Marcos et al., 2008; Jofré et al., 2010). Therefore, the combination of high pressure processing with other hurdles for microbial growth in the food product is needed to warrant a sufficient pathogen reduction and shelf life extension. The use of natural antimicrobials with high pressure processing provides additional food safety assurance while providing a clean label product. Essential oils and bacteriocins from protective cultures have been used in combination with high pressure for improved control of *L. monocytogenes* on RTE meats (Jofré et al., 2008; Marcos et al., 2008; for review, see de Oliveira et al., 2015); however, even the application of hurdle technologies may not reduce cell counts of *L. monocytogenes* by more than 5 log, or prevent re-growth during storage (Marcos et al., 2008; Hereu et al., 2012; de Oliveira et al., 2015). Moreover, past studies did not account for the presence of other meat microbiota that may contribute to spoilage, or to prevention of growth of *L. monocytogenes* during post-treatment refrigerated storage.

Microbiota of refrigerated meat and vacuum-packaged RTE are dominated by *Brochothrix thermosphacta*, *Carnobacterium* spp. and other lactic acid bacteria including psychrotrophic lactobacilli and *Leuconostoc* spp. (Susiluoto et al., 2003; Leisner et al., 2007; Miller et al., 2015). Because RTE products are cooked prior to slicing, package and storage, the microbiota of commercial vacuum packaged RTE products typically has a low diversity (Miller et al., 2015). The extension of the refrigerated storage life of RTE meats by pressure processing requires the control of psychrotrophic spoilage microbiota, or a shift toward growth of microorganisms that do not negatively affect product quality. Reproducible data on pressure effects on non-pathogenic meat microbiota requires experimentation with defined, controlled and reproducible inocula; however, pressure effects on defined strains of meat spoilage organisms or protective cultures *in vitro* or *in situ* are poorly documented (Ulmer et al., 2000; Liu et al., 2012). Moreover, competitive meat microbiota may inhibit growth of *L. monocytogenes*. Inhibition of the growth of *L. monocytogenes* by lactic acid bacteria was often attributed to the production of bacteriocins (Schillinger et al., 1991) but organic acid production by lactic acid bacteria may suffice for inhibition of growth (Bredholt et al., 1999). The role of competitive meat microbiota on post-pressure growth and survival of *L. monocytogenes* has not been systematically explored. Therefore, this study aimed to investigate the impact of pressure, reconstituted non-pathogenic meat microbiota, and antimicrobials on the survival and post-pressure growth of *L. monocytogenes* on RTE ham.

## Materials and Methods

### Bacterial Strains and Growth Conditions

A cocktail of strains containing *L. monocytogenes* FSL J1-177, FSL C1-056, FSL N3-013, FSL R2-499, and FSL N1-227 was used as the “human disease cocktail” recommended for challenge studies in food (Fugett et al., 2006). A cocktail of non-pathogenic RTE meat microbiota contained *B. thermosphacta* FUA3558, *Carnobacterium maltaromaticum* FUA3559, *Leuconostoc gelidum* FUA3560 and FUA3561, and *Lactobacillus sakei* FUA3562. These strains were previously isolated from RTE meats (Miller et al., 2015) and are a reasonable representation of the composition and diversity of microbiota that is recovered from vacuum-packaged and refrigerated RTE meats. *L. monocytogenes* were streaked from -80°C stock cultures onto Tryptic Soy (TS) agar (Difco, Becton Dickinson, Sparks, MD, United States), followed by an overnight subculture in TS broth (TSB), and a second sub-culture with 1% (v/v) inoculum and 16 h incubation. *Listeria* were routinely incubated at 37°C. RTE meat microbiota were prepared in the same manner but grown on All Purpose Tween (APT) agar or broth and incubated at 25°C. For preparation of cocktails, an equal volume of each individual culture was mixed to form a 5-strain cocktail of *L. monocytogenes* or meat microbiota. Strain cocktails were harvested by centrifugation and resuspended in saline (0.85% NaCl) to achieve an optical density at 600 nm of 1.0.

### Preparation of Ham and Antimicrobials

The study used cooked ham with a NaCl concentration of 3% (w/w). The ham was produced experimentally using a product composition and ingredients that match commercial practice in Canada and the United States (Teixeira et al., 2016) Stock solutions of a commercial rosemary extract (NatureGuard^TM^, Newly Weds Foods, Edmonton, AB, Canada) and a 2.5% nisin preparation (>1,000,000 IU/g Sigma-Aldrich, St. Louis, MO, United States) for determination of the minimum inhibitory concentrations were prepared in 50% ethanol and 0.02 N hydrogen chloride (HCl), respectively, to a final concentration of 10% (v/v) and 0.05% (w/v), respectively. Stock solutions of rosemary extract and the 2.5% nisin preparation for surface inoculation of ham were prepared in 0.5% ethanol and 0.02 N HCl, respectively, for a final concentration of 3.3% (v/v) and 175 mg/L, respectively.

### Determination of the Minimal Inhibitory Concentration (MIC) and Minimal Bactericidal Concentrations (MBCs)

Critical dilution assays were used to determine the minimal inhibitory concentration (MIC) and minimal bactericidal concentration (MBC) of rosemary extract and nisin against strains of *L. monocytogenes, B. thermosphacta*, *C. maltaromaticum*, *L. gelidum*, and *L. sakei*. In brief, 100 μl of TS or APT broth was added to each well of a 96 well microtiter plate. Rosemary and nisin stock solutions (100 μl) were added to separate wells and serially diluted across the plate to obtain concentrations ranging from 0.003 to 3% (rosemary extract) and 0.00002 to 0.02% (nisin). Stationary phase cultures of indicator strains were 10-fold diluted in TS or APT broth to a final concentration of about 10^8^ CFU/mL, and microtiter plates were inoculated with 50 μL of diluted cultures. Plates were incubated at 37 (*Listeria*) or 25°C (RTE meat microbiota) for 24 h. To determine the MBC, 100 μl TS or APT broth was pipetted into all wells of a sterile 96 well microtiter plate. After 24 h of incubation of the MIC plates, 10 μl of each well were transferred to a well on the MBC plate. The plates were incubated at the same conditions as the MIC plates. For a better visualization of the bacteria growth in MIC and MBC plates, 40 μl of 0.2 mg/mL p-iodonitrotetrazolium violet (INT; Sigma-Aldrich) was added to each well and incubated for 3 h at 37 (*Listeria* strains) or 25°C (RTE meat microbiota). In the wells that remained colorless after incubation with INT, no bacterial growth was detectable (Eloff, 1998). Experiments were performed in triplicate.

### Sample Preparation and Inoculation for Pressure Treatment

Cooked ham was cut to 20-mm-thick slices with a surface area of 50 cm^2^, vacuum packaged, and stored at 0°C until use. Ham samples were shaped, inoculated and heat-sealed as described (Teixeira et al., 2016). All handling of ham was performed under sterile conditions to prevent microbial contamination, Ham was inoculated with the cocktail of *L. monocytogenes* strains and/or the cocktail of RTE meat microbiota to achieve cell counts of about 10^7^ CFU *Listeria*/g and/or 10^8^ CFU RTE meat microbiota/g. Experimental groups were categorized as follows: (i) *L. monocytogenes*, (ii) RTE meat microbiota, and (iii) *L. monocytogenes* combined with RTE meat microbiota. After inoculation, the ham was placed in Tygon tubing (Tygon S3^TM^ E-3603 Flexible Tubings, Fisherbrand^TM^, Pittsburgh, PA, United States) as described (Teixeira et al., 2016). Each of the three experimental groups were treated with antimicrobials and a solvent control by addition of 20 μL of rosemary extract or 20 μL of nisin to achieve a volumetric concentration exceeding the MIC values against the five strains of *L. monocytogenes* 5- to 20-fold. Antimicrobials were added into the Tygon tubing, the tubing was then heat-sealed and then massaged. Because the ham was surface-inoculated with bacterial strains and the antimicrobial were also added to the surface of the ham, the concentration of antimicrobials was expressed relative to the surface area rather than the volume or weight of the samples. The addition of antimicrobials as described above provided a final surface concentration of ∼325 μg/cm^2^ of rosemary or ∼2 μg/cm^2^ of nisin preparation on the ham samples. To assess the impact of the solvents used for addition of rosemary oil and nisin, solvent controls were prepared by addition of either 20 μL 0.5% ethanol or 20 μL 0.02 N HCl. Samples were maintained at ambient temperature until pressure treatment; after placement of samples in the pressure vessel, the temperature was equilibrated to the treatment temperature for 10 min prior to compression.

### Pressure Treatment

Pressure treatments were carried out in a 2.2 mL high pressure vessel immersed in a temperature-controlled water bath (Teixeira et al., 2016). Initial temperature in the vessel was 5°C and the temperature increase in the pressure vessel during compression was less than 5°C. Ham samples were treated at 500 MPa at 5°C for 1 or 3 min (Teixeira et al., 2016). Detection of surviving cells was determined by surface plating as described below. Additionally, treated samples were stored for 4 weeks at 4°C after treatment, and the presence of viable cells was detected as outlined below. Experiments were performed in triplicate.

### Detection of Surviving Cells

The presence or absence of *L. monocytogenes* and/or RTE microbiota was monitored immediately after pressure treatment and after storage for 4 weeks at 4°C. Untreated samples were analyzed to determine the initial cell count. Un-inoculated ham samples were prepared and stored for 4 weeks at 4°C to ensure the absence of contaminating microbiota from the meat prior the experiment and after storage. Samples were opened aseptically, and the contents were transferred to a sterile 1.5 mL Eppendorf tube and diluted with sterile saline (0.85% NaCl). Ham samples were homogenized for 60 s prior to serial dilutions.

Surviving cells were enumerated by surface plating on selective PALCAM agar (Becton Dickinson) (*L. monocytogenes* plus RTE meat microbiota) and on non-selective TS (*L. monocytogenes*) or APT agar (RTE meat microbiota and *L. monocytogenes* plus RTE meat microbiota). Appropriate dilutions were plated and incubated at 37°C (PALCAM and TS agar) or 25°C (APT agar) for 48 h.

### Extraction of Total DNA and Amplification of Genes Coding for 16S rRNA

DNA was isolated from approximately 20 mg ham by washing of samples with 1 mL saline (0.85% NaCl), followed by centrifugation (5000 × *g* for 10 min) to collect bacterial cells. DNA was extracted from the pellet using DNeasy Blood and Tissue Kit (Qiagen, Toronto, ON, Canada) following the Gram-positive bacteria protocol provided by the manufacturer. Amplicons of the V5–V6 region of 16S rRNA genes obtained with the barcoded primer pair F (5′-GTGCCAGCMGCCGCGGTAA-3′) and R (5′-GGACTACHVGGGTWTCTAAT-3′) (Caporaso et al., 2012) were sequenced on the Illumina MiSeq 2 × 300 bp platform (University of Minnesota Genomics Center; UMGC). Raw sequences were quality filtered with the FastQC tool. Subsequently, the USEARCH pipeline (v9.0.2132, Edgar, 2010) was used to trim and merge pair-end reads, and to sort the sequences into operational taxonomic units (OTUs). OTUs accounting for less than 0.05 % of the total sequences were discarded. Taxonomic classification for each OTU was determined with Ribosomal Database Project (RDP) Classifier 11.1 tool and NCBI database. The relative abundance of bacterial taxa was calculated from three independent replicate samples as percent proportions relative to the total number of sequences in each sample.

### Statistical Analysis

One-way analysis of variance (ANOVA) was performed on SigmaPlot Software to test for significant differences in inhibitory activities of rosemary extract and nisin, and the bactericidal effect of pressure treatments. Significance was assessed with an error probability of 5% (*P* ≤ 0.05). Principle component analysis (PCA) was performed using JMP software (version 13.1.0, SAS Institute Inc., Cary, NC, United States) to examine correlations between gene copies of bacterial groups, pressure treatments and antimicrobials.

## Results

### Inhibitory Activity of Rosemary Extract and Nisin Against *L. monocytogenes* and Competitive Microbiota

Treatments with rosemary extract and nisin were standardized with respect to the antimicrobial activity of these two inhibitors. Therefore, the inhibitory and bactericidal concentrations of rosemary extract and nisin against *Listeria* and RTE meat microbiota were determined. The bactericidal concentration of rosemary extract and nisin was equal or 2- to 3-fold higher than the inhibitory concentration (**Table 1**). Rosemary extract was inhibitory at levels ranging from 1 to 10 g/l; the MIC of nisin ranged from 0.1 to 10 mg/l, corresponding to 2.5–250 μg/l pure nisin. *L. monocytogenes* was significantly more resistant to nisin than other organisms (**Table 1**). Lactic acid bacteria exhibited the highest resistance to rosemary extract and *B. thermosphacta* was most sensitive (**Table 1**).

**Table 1:** MIC and MBC of nisin and rosemary against *L. monocytogenes* and RTE meat microbiota.

Microorganisms	Rosemary extract		Nisin^1^	
	MIC (g/l)	MBC (g/l)	MIC (mg/l)	MBC (mg/l)
*L. monocytogenes* FSL J1-177	3.65 ± 1.04^a^	6.94 ± 2.41^a^	10.42 ± 0.00^a^	10.42 ± 0.00^a^
*L. monocytogenes* FSL R2-499	2.08 ± 0.00^ab^	4.17 ± 0.00^a^	5.21 ± 0.00^ab^	10.42 ± 0.00^a^
*L. monocytogenes* FSL C1-056	2.08 ± 0.00^ab^	4.17 ± 0.00^a^	6.94 ± 3.01^ab^	10.42 ± 0.00^a^
*L. monocytogenes* FSL N1-227	1.82 ± 0.52^b^	5.56 ± 2.41^a^	8.68 ± 3.01^ab^	10.42 ± 0.00^a^
*L. monocytogenes* FSL N3-013	2.60 ± 1.04^ab^	4.17 ± 0.00^a^	3.47 ± 1.50^b^	13.89 ± 6.01^a^
*Brochothrix thermosphacta* FUA3558	1.04 ± 0.00^B^	1.39 ± 0.60^C^	1.52 ± 0.99^AB^	0.87 ± 0.38^B^
*Carnobacterium maltaromaticum* FUA3559	4.17 ± 0.00^A^	3.47 ± 1.20^B^	0.33 ± 0.00^B^	0.87 ± 0.38^B^
*Leuconostoc gelidum* FUA3560	4.17 ± 0.00^A^	4.17 ± 0.00^B^	2.60 ± 0.00^AB^	4.34 ± 1.50^A^
*Leuconostoc gelidum* FUA3561	4.17 ± 0.00^A^	4.17 ± 0.00^B^	3.47 ± 1.50^A^	3.47 ± 1.50^AB^
*Lactobacillus sakei* FUA3562	4.17 ± 0.00^A^	8.33 ± 0.00^A^	0.87 ± 0.38^B^	1.52 ± 0.99^AB^

Data are shown as means ± standard deviations of triplicate independent experiments. Within each column, means with different letters are significantly different (P < 0.05); small letters within Listeria strains, capital letters within competitive organisms. MIC, minimum inhibitory concentrations; MBC, minimum bactericidal concentration. ^1^Indicated in the concentration of the nisin preparation containing 2.5% nisin.

### Effect of Pressure and Reconstituted Microbiota on Survival and Post-pressure Growth of *L. monocytogenes* on Ham

All pressure treatments and storage experiments in this study were performed in aseptically prepared ham containing 3% NaCl. Plating of ham without inoculum confirmed that bacterial contaminants were absent before and after storage (data not shown). The lethality of pressure and antimicrobials was assessed on ham inoculated only with a 5-strain cocktail containing *L. monocytogenes*, a 5-strain cocktail of RTE meat microbiota, or with a combination of both strain cocktails.

### Interactions of Competitive Microbiota and Rosemary Extract on the Lethality of Pressure Against *L. monocytogenes*

**FIGURE 1:**
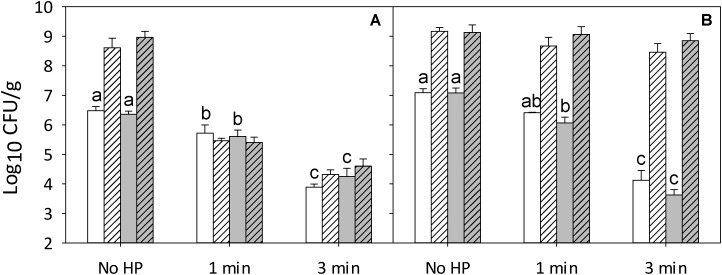
Effect of pressure on survival and post-pressure growth of *L. monocytogenes* and RTE meat microbiota on ham treated with rosemary extract or an equivalent volume of 0.5% ethanol as solvent control. Shown are cell counts of ham inoculated with a 5-strain cocktail of *L. monocytogenes* **(A)** or RTE meat microbiota **(B)** after treatment at 500 MPa for 1 or 3 min without (white) or with (gray) rosemary extract at ∼325 μg/cm^2^. Cell counts of *L. monocytogenes* and other strains were monitored immediately after pressure treatment (solid columns) and after storage for 4 weeks at 4°C (hatched columns). Surviving cells were enumerated by surface plating on non-selective TS (*L. monocytogenes*) or APT agar (RTE meat microbiota). Data are shown as means ± standard deviations of triplicate independent experiments. Treatment means (solid columns) within each panel with different letters are significantly different (*P* < 0.05). The interception point of abscissa and ordinate represents the highest detection limit.

The effect of addition of rosemary extract in 0.5% ethanol on the lethality of pressure was compared to addition of a 0.5% ethanol solution as solvent control (**Figure 1**). Cell counts of control samples containing 0.5% ethanol were comparable to cell counts of control sample without any additives (**Supplementary Figures S1**, **S2**). *L. monocytogenes* counts were reduced by about 1 and 2.5 CFU/g after treatment at 500 MPa for 1 and 3 min, respectively, when ham was inoculated with the *L. monocytogenes* cocktail only (**Figure 1A**). The pressure inactivation of RTE meat microbiota was comparable (**Figure 1B**). Counts of *L. monocytogenes* on pressure-treated samples did not increase during refrigerated storage, while the RTE meat microbiota grew to high cell counts of 10^8^–10^9^ CFU/g during post-pressure refrigerated storage (**Figure 1**).

The addition of rosemary extract did not affect cell counts or growth of *Listeria* or other organisms (**Figure 1**). In combination with pressure at 1 or 3 min, and after 4 weeks of storage at 4°C, rosemary did not affect cell counts of *Listeria* or meat microbiota compared to samples that were subjected to pressure without antimicrobial (**Figure 1**). After 4 weeks of refrigerated storage, all of the samples inoculated with RTE meat microbiota had high cell counts suggesting that rosemary was ineffective in preventing the growth of microorganisms during refrigerated storage (**Figure 1B**).

**FIGURE 2:**
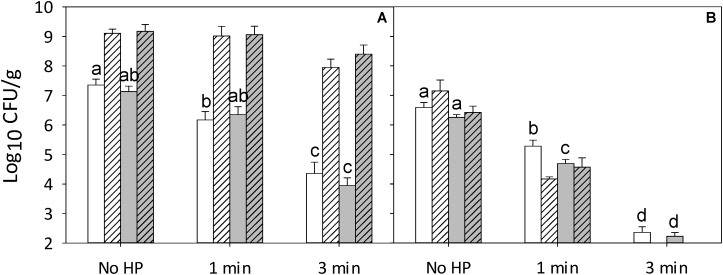
Effect of pressure and RTE meat microbiota on survival and post-pressure growth of *L. monocytogenes* and RTE meat microbiota on ham treated with 0.5% ethanol (solvent control) or an equivalent volume of rosemary extract in 0.5% ethanol. Shown are total cell counts **(A)** on non-selective agar and cell counts of *L. monocytogenes* **(B)** enumerated on selective PALCAM **(B)** agar. Samples were treated at 500 MPa for 1 or 3 min without (white) or with (gray) rosemary at ∼325 μg/cm^2^. Cell counts were enumerated immediately after pressure treatment (solid columns) and after storage for 4 weeks at 4°C (hatched columns). Data are shown as means ± standard deviations of triplicate independent experiments. Treatment means (solid columns) within each panel with different letters are significantly different (*P* < 0.05). The interception point of abscissa and ordinate represents the highest detection limit.

The interaction of RTE meat microbiota and antimicrobials on the lethality of pressure was further investigated on ham that was inoculated with both strain cocktails, the *Listeria* cocktail and the RTE meat microbiota. The total cell counts of ham that was inoculated with both cocktails and treated at 500 MPa were comparable to the cell counts of pressure treated ham with RTE meat microbiota only (**Figures 1B**, **2A**). After 4 weeks of refrigerated storage, RTE microbiota grew to high cell counts (**Figure 2A**) irrespective of the addition of rosemary extract. Growth of RTE meat microbiota inhibited the growth of *L. monocytogenes* regardless of the presence of rosemary extract (**Figure 2B**). Treatment of ham with 500 MPa for 3 min in presence of RTE meat microbiota reduced cell counts of *Listeria* to levels below the detection limit after 4 weeks of storage (**Figure 2B**). Of note, the enumeration of *L. monocytogenes* on selective PALCAM agar immediately after pressure treatment may not accurately reflect total cell counts because sublethally injured cells are not accounted for. However, 4 weeks of refrigerated storage suffice to repair sublethal injury in the psychrotrophic *L. monocytogenes* and cell counts on selective PALCAM agar after 4 weeks of storage thus accurately reflect total cell counts (Teixeira et al., 2016).

### Interactions of RTE Meat Microbiota and Nisin on the Lethality of Pressure

**FIGURE 3:**
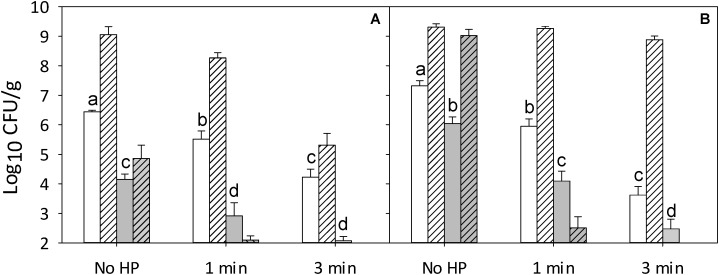
Effect of pressure on survival and post-pressure growth of *L. monocytogenes* and RTE meat microbiota on ham. Ham was inoculated with a 5-strain cocktail of *L. monocytogenes* **(A)** or RTE meat microbiota **(B)** and treated at 500 MPa for 1 or 3 min with 0.02 N HCl (solvent control) or an equivalent volume of a nisin preparation in 0.02 N HCl to achieve a concentration of 2 μg/ cm^2^, corresponding to 0.05 μg/cm^2^ pure nisin. Cell counts of *L. monocytogenes* and RTE meat microbiota were monitored immediately after pressure treatment (solid columns) and after storage for 4 weeks at 4°C (hatched columns). Surviving cells were enumerated on non-selective TS (*L. monocytogenes*) or APT agar (other strains). Data are shown as means ± standard deviations of triplicate independent experiments. Treatment means (solid columns) within each panel with different letters are significantly different (*P* < 0.05). The interception point of abscissa and ordinate represents the highest detection limit.

The effect of nisin in 0.02 N HCl on the lethality of pressure was compared to addition of 0.02 N HCl as solvent control (**Figure 3**). Cell counts of control samples containing 0.02 N HCl were comparable to cell counts of control samples containing 0.5% ethanol (**Figure 1**) or samples without any additive other than the inoculum (**Supplementary Figure S1**).

The combined effect of pressure and nisin was evaluated by treatments at 500 MPa for 1 and 3 min. Nisin reduced cell counts by 2 log (CFU/g) without pressure treatment but did not prevent growth of *L. monocytogenes* during refrigerated storage (**Figure 3A**). In combination with pressure treatment for 3 min, nisin increased the inactivation of *L. monocytogenes* by more than 2 log (CFU/g) compared to samples subjected to pressure alone, and reduced counts of *L. monocytogenes* to levels below the detection limit of 2 log (CFU/g) after 28 days of storage at 4°C (**Figure 3A**). RTE meat microbiota were more resistant to nisin than *L. monocytogenes* (**Figure 3B**). After 4 weeks of refrigerated storage, untreated samples inoculated with RTE meat microbiota had high cell counts, suggesting that nisin alone did not prolong the storage life (**Figure 3B**). In combination with a 3 min pressure treatment, nisin reduced cell counts of RTE meat microbiota to levels below the detection limit of 2 log (CFU/g) after 28 days of storage at 4°C (**Figure 3B**).

**FIGURE 4:**
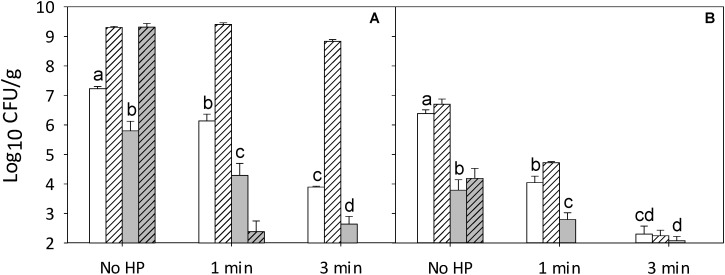
Effect of pressure and RTE meat microbiota on survival and post-pressure growth of *L. monocytogenes* and RTE meat microbiota on ham treated with 0.02 N HCl (solvent control) or an equivalent volume of a nisin preparation in 0.02 N HCl to achieve a concentration of 2 μg/cm^2^, corresponding to 0.05 μg/cm^2^ pure nisin. Shown are total cell counts **(A)** on non-selective agar and cell counts of *L. monocytogenes* **(B)** enumerated on selective PALCAM **(B)** agar. Samples were treated at 500 MPa for 1 or 3 min without (white) or with (gray) nisin. Cell counts ere enumerated immediately after pressure treatment (solid columns) and after storage for 4 weeks at 4°C (hatched columns). Data are shown as means ± standard deviations of triplicate independent experiments. Treatment means (solid columns) within each panel with different letters are significantly different (*P* < 0.05). The interception point of abscissa and ordinate represents the highest detection limit.

The interaction of competitive microbiota and nisin on the lethality of pressure was further investigated on ham surface-inoculated with *Listeria* and RTE meat microbiota. The cocktail of RTE meat microbiota alone prevented growth of *Listeria* refrigerated storage even without pressure treatment or addition of nisin (**Figure 4B**). Pressure effects on RTE meat microbiota were comparable to those effects that were observed with ham containing RTE meat microbiota only (**Figures 3B**, **4A**). After 4 weeks of refrigerated storage, total cell counts exceeded 8 log (CFU/g) (**Figure 4A**). Pressure treatment of ham for 1–3 min eliminated *Listeria* after 4 weeks of storage when RTE meat microbiota were also present (**Figure 4B**). The effect of nisin on total cell counts of ham containing both strain cocktails was comparable to the effect of nisin ham inoculated with RTE meat microbiota only (**Figures 3B**, **4B**). Nisin in combination with pressure reduced cell counts to less than 3 log (CFU/g) and prevented growth during refrigerated storage (**Figure 4A**). Combination of nisin with 1 or 3 min pressure treatment in presence of RTE meat microbiota reduced cell counts of *L. monocytogenes* to levels below the detection limit after 4 weeks of refrigerated storage (**Figure 4B**). The presence of non-pathogenic meat microbiota thus not only inhibited growth of *L. monocytogenes* even without pressure or antimicrobials, but also increased the efficacy of pressure and antimicrobial compounds.

### Composition of Ham Microbiota Assessed by High Throughput Sequencing of 16S rRNA Sequence Tags

The cocktail of RTE meat microbiota contains species that differ with respect to their resistance to pressure and antimicrobials, and with respect to their impact on product quality. To characterize the composition of ham microbiota after pressure processing and refrigerated storage, 16S rRNA tags were sequenced by paired end Illumina sequencing. The average and median number of reads per sample after quality control and trimming was 34,000 reads per sample; the relative abundance of bacterial taxa is reported as average ± standard deviation of three independent replicates (**Table 2**). The microbiota of ham without additions matched the microbiota of control ham with addition of 0.5% ethanol or 0.02 N HCl (**Table 2** and **Supplementary Table S1**). Sequences matching species present in the inoculum accounted for more than 99.9% of sequences in most samples, demonstrating that the aseptic preparation of ham achieved control of ham microbiota throughout sample processing and storage (**Table 2** and **Supplementary Table S1**). The only exceptions pertain to ham that was pressure treated in presence of nisin. In these samples, total cell counts were below 3 log (CFU/g) and sequencing of 16S rRNA sequence tags likely also accounted for DNA of dead bacterial cells that carried over from raw material used for ham processing. In keeping with the cell count data, sequences matching *L. monocytogenes* were below 1% of sequences in all samples. Again, exceptions pertain to ham that was pressure treated in presence of nisin; here, the relative abundance of bacterial DNA reflects the proportion of species in the inoculum and sequences matching *L. monocytogenes* accounted for about 10% of sequences (**Table 2**).

**Table 2:** Relative abundance (%) of 16S rRNA gene sequences from DNA isolated from ham after treatment at 500 MPa for 1 or 3 min, followed by storage for 4 weeks at 4°C.

Controls	0.5% EtOH						HCl					
Samples	Meat microbiota			*Listeria* and meat microbiota			Meat microbiota			*Listeria* and meat microbiota		
				
Treatments	No HP	1 min	3 min	No HP	1 min	3 min	No HP	1 min	3 min	No HP	1 min	3 min
**Organism (%)**												
***Brochothrix***	45.1 ± 3.5	72.3 ± 11.5	81.5 ± 14.7	47.0 ± 1.0	75.9 ± 10.1	93.6 ± 4.0	51.6 ± 2.7	71.7 ± 16.2	75.6 ± 4.7	50.5 ± 5.2	58.4 ± 8.8	76.8 ± 10.0
***Carnobacterium***	18.7 ± 0.2	3.1 ± 2.6	12.6 ± 10.6	17.5 ± 4.1	2.7 ± 2.0	0.68 ± 0.34	14.6 ± 1.7	3.45 ± 1.28	9.17 ± 2.84	14.0 ± 0.83	4.65 ± 4.88	8.81 ± 6.29
***Leuconostoc***	23.8 ± 0.26	23.0 ± 13.2	1.28 ± 0.83	21.9 ± 11.0	19.6 ± 12.3	4.8 ± 4.8	25.5 ± 4.7	23.2 ± 14.6	11.4 ± 3.3	26.4 ± 5.5	31.0 ± 10.0	10.5 ± 4.5
***Lactobacillus***	12.1 ± 3.5	1.61 ± 0.86	4.51 ± 3.30	9.44 ± 1.28	1.26 ± 0.62	0.56 ± 0.30	8.11 ± 0.84	1.54 ± 0.56	3.70 ± 1.16	8.21 ± 0.39	1.93 ± 1.86	3.40 ± 2.02
***Listeria***	0.08 ± 0.01	0.02 ± 0.01	0.05 ± 0.01	0.64 ± 0.25	0.42 ± 0.36	0.26 ± 0.14	0.04 ± 0.01	0.03 ± 0.01	0.03 ± 0.02	0.80 ± 0.23	0.49 ± 0.26	0.32 ± 0.26
**Others**	–	–	–	–	–	–	–	–	–	–	–	–

**Antimicrobials**	**Rosemary extract**						**Nisin**					
**Samples**	**Meat microbiota**			***Listeria* and meat microbiota**			**Meat microbiota**			***Listeria* and meat microbiota**		
				
**Treatments**	**No HP**	**1 min**	**3 min**	**No HP**	**1 min**	**3 min**	**No HP**	**1 min**	**3 min**	**No HP**	**1 min**	**3 min**

**Organism (%)**												
***Brochothrix***	30.7 ± 12.6	56.2 ± 23.7	68.1 ± 24.0	37.0 ± 5.9	74.8 ± 30.2	84.9 ± 15.1	4.8 ± 2.9	21.1 ± 4.4	20.3 ± 3.1	7.1 ± 5.4	15.9 ± 2.0	20.9 ± 1.7
***Carnobacterium***	22.6 ± 1.9	3.64 ± 3.18	3.33 ± 3.00	24.3 ± 5.0	1.85 ± 1.97	1.82 ± 2.01	3.68 ± 1.42	19.5 ± 0.7	19.3 ± 0.8	4.90 ± 3.46	16.5 ± 1.9	16.2 ± 1.1
***Leuconostoc***	32.6 ± 11.5	38.1 ± 24.8	26.6 ± 26.0	25.3 ± 4.0	21.4 ± 26.2	11.3 ± 11.6	85.8 ± 6.00	21.1 ± 3.3	27.4 ± 6.3	75.0 ± 16.2	22.8 ± 7.3	19.8 ± 5.3
***Lactobacillus***	13.7 ± 2.4	1.69 ± 0.56	1.75 ± 0.93	12.7 ± 1.1	1.11 ± 1.24	1.45 ± 1.71	3.44 ± 1.65	22.9 ± 5.0	18.6 ± 4.1	5.32 ± 4.12	18.3 ± 5.2	17.3 ± 5.2
***Listeria***	0.03 ± 0.02	0.05 ± 0.03	0.02 ± 0.01	0.59 ± 0.09	0.62 ± 0.85	0.38 ± 0.06	0.01 ± 0.00	0.21 ± 0.15	0.06 ± 0.00	3.11 ± 1.93	10.8 ± 4.5	12.8 ± 6.5
**Others**	–	–	–	–	–	–	1.96 ± 0.10	13.6 ± 4.9	12.9 ± 7.5	3.72 ± 1.71	13.8 ± 7.8	10.9 ± 2.4

Data are shown as means ± standard deviations of triplicate independent experiments.

The predominant species in untreated ham after 4 weeks were *B. thermosphacta, C. maltaromaticum*, and *L. gelidum*; *L. sakei* was less abundant. The use of rosemary extract did not influence the composition of meat microbiota. Pressure treatment increased the relative abundance of *B. thermosphacta* and nisin shifted the composition of ham microbiota toward a higher abundance *L. gelidum*.

### Multivariate Data Analysis of the Composition of Ham Microbiota After Storage

**FIGURE 5:**
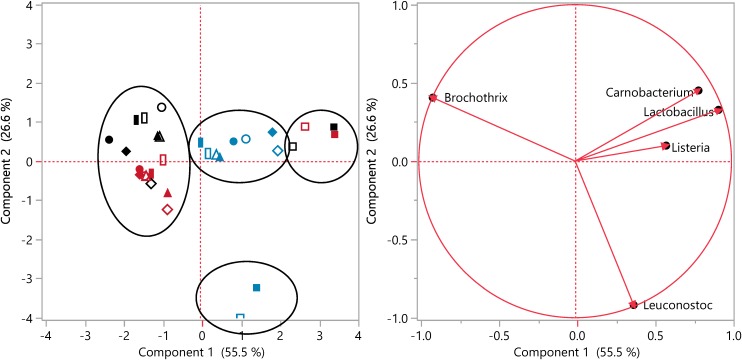
Principal component analysis (PCA) of the % abundance of 16S rRNA sequences on ham containing a nisin preparation, rosemary extract, or without additions after storage for 4 weeks at 4°C. Ham was inoculated with 5-strain cocktails of *L. monocytogenes* and RTE meat microbiota, treated for 1 or 3 min at 500 MPa, or left untreated. The percentage of variation explained by the plotted principal coordinates is indicated on the axes. Each symbol represents a type of sample and each color represents a treatment: no addition (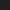), or controls with 0.5% ethanol (•) or 0.02N HCl (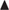), rosemary extract (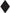), nisin (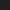); no pressure treatment (blue), 1 min at 500 MPa (red), 3 min at 500 MPa (black).

The relationship between the composition of meat microbiota, pressure treatment, and addition of antimicrobials was further assessed using principal component analysis (PCA) (**Figure 5**). The loading plot separated samples into four main clusters comprising pressure treated ham, untreated ham, ham prepared with nisin, and pressure treated ham prepared with nisin (**Figure 5**). The loading plot of individual bacterial taxa demonstrated that the separate clustering of pressure treated ham related to the high abundance of *B. thermosphacta;* separate clustering of nisin-treated samples related to a high abundance of *L. gelidum*. The abundance of bacterial species in pressure treated ham containing nisin essentially represents the inoculum prior to pressure treatment and their location in the linear discriminant analysis plot thus relates to *Carnobacterium*, *Lactobacillus*, and *Listeria*.

## Discussion

Pressure processing of RTE meats at cold or ambient temperatures does not consistently result in a 5-log reduction of *L. monocytogenes* and surviving cells are able to grow during subsequent refrigerated storage (Jofré et al., 2010; Teixeira et al., 2016). This is not satisfactory for the requirements of the industry to guarantee product safety and extended storage life. An alternative approach to inhibit *L. monocytogenes* in RTE meats comprises the combination of high pressure and natural antimicrobials (Marcos et al., 2008; Hereu et al., 2012; de Oliveira et al., 2015). This study assessed the impact of pressure, RTE meat microbiota, and antimicrobials on survival and growth of *L. monocytogenes* on ready-to-eat ham. The ham was custom produced for use in challenge studies to obtain a product that matches commercial RTE ham but is free of preservatives or other antimicrobials (Liu et al., 2014; Teixeira et al., 2016). Aseptic preparation of ham slices for pressure treatment allowed control of microbiota throughout treatment and 4 weeks of storage. Products were inoculated with a 5 strain cocktail of non-pathogenic ham microbiota containing *B. thermosphacta*, *L. gelidum*, *C. maltaromaticum* and *L. sakei* to obtain a defined and controlled inoculum, and to match the diversity and composition of microbiota on vacuum packaged RTE meat products that are stored at refrigeration temperature (Susiluoto et al., 2003; Leisner et al., 2007; Miller et al., 2015). After storage, sampled contained only those organisms that were used as inoculum, i.e., *L. monocytogenes* and/or RTE meat microbiota. In addition to culture dependent analysis of ham microbiota after pressure treatment and storage, microbiota were analyzed by high throughput sequencing of 16S rRNA sequence tags, which has become a valuable tool for analysis of meat microbiota (Pothakos et al., 2014; Fougy et al., 2016; Säde et al., 2017). Taken together, the experimental approach allowed control of product composition, process parameters, and microbiota throughout processing and storage.

Rosemary essential oil is used commercially in meat products to prevent lipid oxidation (Estévez and Cava, 2006); rosemary essential oil also has antimicrobial effects against *L. monocytogenes* and demonstrated synergistic effects with pressure application in buffer (Espina et al., 2013; Abdollahzadeh et al., 2014). Our findings confirmed antimicrobial activity of rosemary essential oil against *L. monocytogenes* but rosemary oil did not affect growth and survival of *L. monocytogenes* in ham or pressure treated ham during refrigerated storage. The meat matrix may protect bacteria against the inhibitory activity of spice extracts (Zhang et al., 2009). The strain-dependent variation of nisin sensitivity of observed in this study was consistent with prior reports that employed 200 strains of *L. monocytogenes* and reported an MIC range of 0.002–0.8 mg pure nisin/L (Katla et al., 2003), corresponding to 0.08–32 mg/L of the nisin preparation employed in the present study. Also consistent with prior reports, the combination of nisin and pressure in RTE ham displayed an additive effect against *L. monocytogenes* and prevented post-pressure resuscitation of sublethally injured cells (Jofré et al., 2008; Hereu et al., 2012). Surface treatment of ham, however, may not be economically feasible and nisin is inactivated in raw meat (Rose et al., 1999). Other bacteriocins of lactic acid bacteria, particularly Listeria-active class IIa bacteriocins, have a comparable mode of action, are compatible with use on raw or processed meats, and exert a comparable synergistic effect with high pressure on *L. monocytogenes* in ham (Garriga et al., 2002; Marcos et al., 2008).

Competitive meat microbiota consisting of lactic acid bacteria inhibit growth of *L. monocytogenes* in meat products; however, their resistance to pressure is poorly documented (Bredholt et al., 1999; Liu et al., 2012). Differential inactivation of lactic acid bacteria and *Listeria* on meat by pressure and/or antimicrobials may enhance growth of *L. monocytogenes* if the antibacterial intervention inactivates competitive microbiota while few cells of *L. monocytogenes* survive. To date, differential inactivation of lactic acid bacteria and *Listeria* has only been evaluated with cheese starter cultures (Arqués et al., 2005). Our study demonstrated that reconstitution with a 5-strain cocktail of RTE meat microbiota inhibited growth of *L. monocytogenes* during refrigerated storage. The pressure resistance of RTE meat microbiota was equivalent to *L. monocytogenes*; remarkably, the combination of reconstituted meat microbiota with pressure treatment of 3 min reduced levels of *L. monocytogenes* by more than 5 log (CFU/g). This results contrasts recovery and growth of *L. monocytogenes* after pressure treatment without competitive microbiota (this study, Jofré et al., 2008; Hereu et al., 2012) and may allow the design of pressure processes for control of *Listeria* with protective cultures but without use of additional antimicrobial hurdles.

Meat microbiota are differentially inactivated by bacteriocins of lactic acid bacteria (Balay et al., 2017); this may impact product quality because different organisms differentially affect the sensory properties of meat products. *B. thermosphacta* causes off-odors already at relatively low cell counts (Vermeiren et al., 2005), *L. gelidum* and other psychrotrophic *Leuconostoc* spp. cause spoilage through production of CO_2_, and by exopolysaccharide production from sucrose (Vermeiren et al., 2005; Pothakos et al., 2014). In comparison, growth of *L. sakei* or *C. maltaromaticum* on RTE meats has only limited impact on sensory properties (Vermeiren et al., 2005; Leisner et al., 2007). *L. sakei* and *C. maltaromaticum* were least resistant to treatments with pressure and/or nisin. The addition of nisin consistently selected for growth of *L. gelidum* while pressure treatments favored growth of *B. thermosphacta*. Antimicrobial interventions aiming at control of *L. monocytogenes* also shifted the composition of other meat microbiota and may result in a more prominent spoilage phenotype (Vermeiren et al., 2005; Leisner et al., 2007) unless microbiota are controlled by process hygiene and/or protective cultures. *C. maltaromaticum*, which is used commercially as a protective culture on meat products and seafood (Balay et al., 2017; Saraoui et al., 2017), may not be suitable for use in combination with pressure treatment.

## Conclusion

This study evaluated the use of pressure in combination with antimicrobials for control of *L. monocytogenes* in a meat system with controlled and reconstituted meat microbiota. Consistent with prior reports, pressure alone was insufficient for control of *L. monocytogenes*. Pressure treatment in presence of reconstituted meat microbiota; however, reduced cell counts of *L. monocytogenes* by more than 5 log (CFU/g) and cell counts were reduced further to levels below the detection limit after 4 weeks of refrigerated storage. This finding demonstrates that competitive meat microbiota inhibit recovery of sub-lethally injured *L. monocytogenes* after pressure treatment. Rosemary essential oil did not improve control of *Listeria* by pressure and/or competitive meat microbiota but nisin additionally decreased cell counts of *L. monocytogenes.* Competitive organisms displayed higher resistance to nisin than *L. monocytogenes* and nisin alone did not extend the storage life of products. Nisin and pressure application, however, resulted in characteristic shifts of meat microbiota which may alter the spoilage phenotype of products. Overall, the study supports earlier reports (Arqués et al., 2005; Balay et al., 2017) that competitive microbiota are an important determinant of the survival of *L. monocytogenes* in ready-to-eat foods and their presence should be considered in challenge trials aiming at improved *Listeria* control.

## Author Contributions

JT, MG, and LM designed the experiments. JT and LR conducted the experiments. JT and MG drafted the figures and tables. JT, MG, and LM wrote the manuscript.

## Conflict of Interest Statement

The authors declare that the research was conducted in the absence of any commercial or financial relationships that could be construed as a potential conflict of interest. The reviewer GS and handling Editor declared their shared affiliation at the time of review.
