# Supplementary material for: Effect of Pressure, Reconstituted RTE Meat Microbiota, and Antimicrobials on Survival and Post-pressure Growth of Listeria monocytogenes on Ham
Source: Front Microbiol. 2018 Aug 27;9:1979. doi: 10.3389/fmicb.2018.01979 (PMC6119701; doi:10.3389/fmicb.2018.01979)
Supplement: Supplementary file 1 [file Data_Sheet_1.pdf]

Effect of pressure, meat microbiota, and antimicrobials on survival and post-pressure growth of *Listeria monocytogenes* on ham

Januana S. Teixeira, Lenka Repková, Lynn M. McMullen, Michael G. Gänzle§

University of Alberta, Department of Agricultural, Food and Nutritional Science, Edmonton, Canada

Online supplementary material.

Table S1. Relative abundance (%) of *L. monocytogenes* and reconstituted meat microbiota on ham after high-pressure treatment at 500 MPa for 1 or 3 min following storage for 4 weeks at 4 °C.

Figure S1. Effect of pressure on survival and post-pressure growth of *L. monocytogenes* or reconstituted microbiota on ham (inoculum only).

Figure S2. Effect of pressure and reconstituted meat microbiota on survival and post-pressure growth of *L. monocytogenes* on ham (inoculum only).

Table S1. Relative abundance (%) of *L. monocytogenes* and non-pathogenic meat microbiota on ham after high-pressure treatment at 500 MPa for 1 or 3 min following storage for 4 weeks at 4 °C. Data are shown as means ± standard deviations of triplicate independent experiments.

**Samples**	**Meat Microbiota**			***Listeria* and Meat Microbiota**		
**Treatments**	**No HP**	**1 min**	**3 min**	**No HP**	**1 min**	**3 min**
**Organisms**						
Brochothrix	51.57±2.65	71.68±16.15	75.62±4.72	50.54±5.16	58.40±8.81	76.78±10.04
Carnobacterium	14.58±1.73	3.45±1.28	9.17±2.84	14.01±0.83	4.65±4.88	8.81±6.29
Leuconostoc	25.54±4.70	23.21±14.63	11.35±3.33	26.35±5.54	31.01±9.98	10.51±4.54
Lactobacillus	8.11±0.84	1.54±0.56	3.70±1.16	8.21±0.39	1.93±1.86	3.40±2.02
Listeria	0.04±0.01	0.03±0.01	0.03±0.02	0.80±0.23	0.49±0.26	0.32±0.26

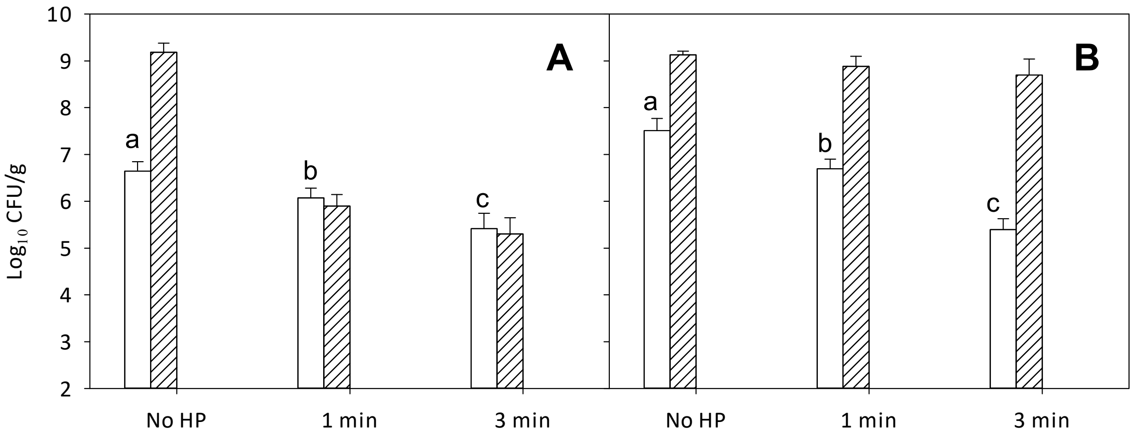

Figure S1. Effect of pressure on survival and post-pressure growth of *L. monocytogenes* or competitive microbiota on ham (inoculum only). Shown are the total CFU/g of ham inoculated with *L. monocytogenes* (A) and reconstituted meat microbiota (B) with or without pressure treatment at 500 MPa for 1 or 3 min. The presence or absence of *L. monocytogenes* and competitive microbiota was monitored immediately after pressure treatment (solid columns) and after storage for 4 weeks at 4 °C (patterned columns). Surviving cells were enumerated by surface plating on nonselective TS (*L. monocytogenes*) or APT agar (competitive microbiota) for the determination of viable and sublethally injured cells. Appropriate dilutions were plated and incubated at 37 (TS agar) or 25 °C (APT agar) for 48 h. Data are shown as means ± standard deviations of triplicate independent experiments. Treatment means (solid columns) within each panel with different letters are significantly different ($P < 0.05$). The interception point of abscissa and ordinate represents the highest detection limit.

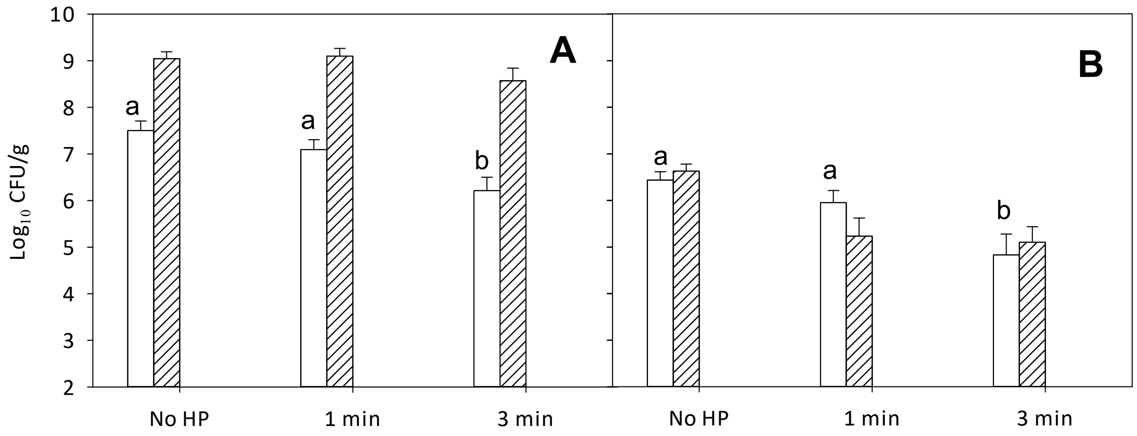

Figure S2. Effect of pressure and reconstituted meat microbiota on survival and post-pressure growth of *L. monocytogenes* on ham (inoculum only). Cell counts were enumerated nonselective TS agar (**Panel A**) and on PALCAM agar selective for *L. monocytogenes* (**Panel B**) in untreated samples or after pressure treatment at 500 MPa for 1 or 3 min. Cell counts were monitored immediately after pressure treatment (solid columns) and after storage for 4 weeks at 4 °C (patterned columns). Appropriate dilutions were plated and incubated at 37 °C for 48 h. Data are shown as means ± standard deviations of triplicate independent experiments. Treatment means (solid columns) within each panel with different letters are significantly different ($P < 0.05$). The interception point of abscissa and ordinate represents the highest detection limit.
